# How to assemble a beneficial microbiome in three easy steps

**DOI:** 10.1111/j.1461-0248.2012.01853.x

**Published:** 2012-08-22

**Authors:** István Scheuring, Douglas W Yu

**Affiliations:** 1 Research Group in Theoretical Biology and Evolutionary Ecology, Department of Plant Systematics, Ecology and Theoretical Biology, Eötvös University and HAS, Pázmány P. sétány 1/C, H-1117, Budapest, Hungary; 2 State Key Laboratory of Genetic Resources and Evolution, Kunming Institute of Zoology, Chinese Academy of Sciences, Kunming, Yunnan, 650223, China; 3 School of Biological Sciences, University of East Anglia, Norwich Research Park, Norwich, Norfolk, NR47TJ, UK

**Keywords:** Actinomycetes, antibiotics, Attini, game theory, horizontal transmission, microbiome, mutualism, screening, *S treptomyces*, symbiosis

## Abstract

There is great interest in explaining how beneficial microbiomes are assembled. Antibiotic-producing microbiomes are arguably the most abundant class of beneficial microbiome in nature, having been found on corals, arthropods, molluscs, vertebrates and plant rhizospheres. An exemplar is the attine ants, which cultivate a fungus for food and host a cuticular microbiome that releases antibiotics to defend the fungus from parasites. One explanation posits long-term vertical transmission of *P seudonocardia* bacteria, which (somehow) evolve new compounds in arms-race fashion against parasites. Alternatively, attines (somehow) selectively recruit multiple, non-coevolved actinobacterial genera from the soil, enabling a ‘multi-drug’ strategy against parasites. We reconcile the models by showing that when hosts fuel interference competition by providing abundant resources, the interference competition favours the recruitment of antibiotic-producing (and -resistant) bacteria. This partner-choice mechanism is more effective when at least one actinobacterial symbiont is vertically transmitted or has a high immigration rate, as in disease-suppressive soils.

We know it works in practice, but does it work in theory?

-An old joke about bureaucrats and economists

## INTRODUCTION

Explaining how microbiomes are established and maintained is now one of the leading questions in biology and has attracted investigators from molecular biology to community ecology to evolutionary game theory (Prosser *et al*. 2007; Chaston & Goodrich-Blair 2010; Pepper & Rosenfeld 2012). Host species receive a multitude of benefits from their microbial communities, such as enhanced nutrition and protection from enemies, and it is increasingly accepted that hosts assemble non-random sets of microbial symbionts and that these sets have higher frequencies of beneficial symbionts than expected by chance (Dethlefsen *et al*. 2007; Kaltenpoth 2009; Mendes *et al*. 2011; Seipke *et al*. 2012b). In other words, hosts appear to be able to choose the right microbial partners (sometimes only a single species) out of a huge pool of candidates, but a detailed understanding of the evolution of successful partner choice remains unclear. How a host assembles a beneficial microbiome is the central question of our study.

It can be useful to think about microbiome biology as a branch of community ecology (Prosser *et al*. 2007; Konopka 2009; Pepper & Rosenfeld 2012), just with different vocabulary. For instance, gut microbiomes are shaped by priority effects (vertical transmission, e.g. Ochman *et al*. 2010), resources (diets, e.g. Muegge *et al*. 2011), immigration (bacterial transplantation, e.g. Manichanh *et al*. 2010) and disturbance (antibiotics, e.g. Dethlefsen & Relman 2010).

However, there is also a profound difference with community ecology in that for a microbiome, *the environment is itself a host species that evolves*, potentially in ways that affect the composition of the microbiome. Not surprisingly, there is evidence that gut microbiome composition is governed by host species (Ochman *et al*. 2010) and, more suggestively, by host genotype (Benson *et al*. 2010).

To model the coevolution of host and microbiome, Archetti (2011) and Archetti *et al*. (2011a,b) introduced the concept of *screening* from economic game theory. If the host can pair its reward to symbionts with a ‘demanding environment’, such that the combination turns out to be attractive to mutualists and unattractive to parasites, selective recruitment of mutualists occurs, even if the host is unable to differentiate symbionts. The host does not choose, and never needs to know, the quality of any individual symbiont; if the host evolves to set living conditions correctly, the potential symbionts evolve to accept the host or to reject it (and remain free living), according to each symbiont's type.

Our purpose here is to identify conditions under which hosts can successfully ‘screen-in’ microbiomes that are dominated by antibiotic-producing (and -resistant) bacteria, of which there is a fast-growing number of examples, ranging across arthropods (especially insects), plant rhizospheres, marine corals, sponges, cone snails and even the hoopoe bird (Ritchie 2006; Taylor *et al*. 2007; Soler *et al*. 2008; Kaltenpoth 2009; Mendes *et al*. 2011; Seipke *et al*. [Bibr b43 b44]). Given the prevalence of plants, corals, and arthropods, it is arguable that this type of microbiome is the most abundant microbiome in nature.

To identify assembly conditions, we will use *competition-based screening*, in which the demanding environment derives from competition amongst the symbionts (Archetti *et al*. 2011b); if a host can foment competition in such a way that beneficial symbionts have competitive superiority, symbionts evolve to screen themselves in or out, depending on their type, or non-beneficial symbionts are simply competitively excluded.

Also, to ground the model empirically, we will focus on one system, the cuticular microbiome of the attine ants (Hymenoptera, Formicidae, Attini), which is by far the best studied of its kind (Caldera *et al*. 2009; Barke *et al*. 2011). Attine ants live in a mutualistic symbiosis with a vertically transmitted fungus and with antibiotic-producing actinomycetes. The ants house the fungus in a warm and humid environment and feed it plant matter (the two most derived genera are known as ‘leafcutters’), and, in return, the fungus digests the plant material to feed the ant colony. The ants also house, and apparently feed (Currie *et al*. 2006), their bacteria, which in return provide antibiotics that the ants use to kill pathogenic fungi and bacteria that invade the fungal gardens.

The longstanding explanation of the system is that only bacteria in the genus *Pseudonocardia* (Actinobacteria, Actinomycetales) are true mutualists, having coevolved and codiversified with the attine ants via vertical transmission, with rare recruitment across ant lineages or from the environment (Currie *et al*. 1999; Cafaro *et al*. 2011). However, this interpretation raises the difficult (and exciting) problem of explaining how antibiotic efficacy has been maintained over millions of years, given the constant presence of both generalist and specialist fungal pathogens, such as the mould *Escovopsis*. The competing explanation is that attine ants constantly recruit a diverse range of actinobacteria from the soil environment, which maintains efficacy via the simultaneous application of antibiotics with different modes of action. In fact, a number of recent studies have shown that attine cuticle microbiomes contain a variety of actinobacterial species, and some of those studies have shown that those bacteria produce antibacterials and antifungals in culture (Kost *et al*. 2007; Mueller *et al*. 2008,^2010^; Haeder *et al*. 2009; Sen *et al*. 2009; Barke *et al*. 2010,^2011^; Andersen 2011; Schoenian *et al*. 2011; Seipke *et al*. 2011; Mueller 2012). One notable study has demonstrated that multiple *Acromyrmex echinatior* workers that were collected off a fungal garden host a *Streptomyces* species that produces valinomycin *in vivo*, on worker integuments (Schoenian *et al*. 2011).

However, the presence of multiple bacterial mutualists raises two new problems: How does the ant selectively take up beneficial bacteria out of the enormous bacterial pool in the soil? And how is diversity maintained within the microbiome, given competition for the ant niche?

Sen *et al*. (2009) have suggested that antibiotic production may be selected for if it confers competitive superiority for the attine-ant niche, meaning that antibiotic activity against pathogens can be thought of as a by-product of bacterial interference competition. (Interference competition means that one species directly harms another). A competition-based screening interpretation of Sen *et al*.'s idea would further hypothesise that the ant host has evolved to favour interference competitors over exploitation competitors (e.g. fast-growers) by providing the right level and mix of resources to fight over. Consistent with this scenario, *Pseudonocardia* strains isolated from attine colonies fight each other *in vitro* (Poulsen *et al*. 2007), and antibiotic compounds produced by *Acromyrmex*-isolated *Streptomyces* can kill other attine-associated bacteria *in vitro*, including *Pseudonocardia* (Schoenian *et al*. 2011).

We now model competition-based screening and show that (1) hosts can confer competitive superiority on antibiotic-producing (and -resistant) bacteria by providing high levels of resources, and that (2) such microbiomes exhibit priority effects, so fixation of antibiotic-producers in the microbiome is more likely if antibiotic-producers are more abundant than non-producers at initial colonisation. Thus, vertical transmission of one antibiotic-producing lineage (or a higher rate of immigration from the environment) can promote the horizontal recruitment of other antibiotic-producing species, thereby reconciling the two competing theories of the attine cuticular microbiome.

## MODEL

It is convenient to start with Mao-Jones *et al*.'s (2010) strategic model of a coral microbiome, which has been used to study the dynamics of coral bleaching as a consequence of disturbance, host resource provisioning, and interference competition. Their model has four variables: substrate (*S*), beneficial microbes (*B*), antibiotics (*A*) and pathogenic microbes (*P*). *B* and *P* can represent individual species or functional groups of bacteria, and the terms are used to differentiate antibiotic producers from non-producers respectively. Pathogens are therefore parasitic in the sense that they consume host resources but provide no benefits, thereby imposing opportunity costs on the host. Note that Beneficials are always resistant to their own antibiotics, or antibiotic production would be suicidal, and Beneficials are typically also resistant to many antimicrobials that they do not themselves produce (Wright 2010). Thus, only Beneficials can directly harm Pathogens, and not the other way around

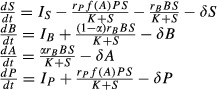
(1)

*I*_*S*_ is the substrate production rate, *I*_*P*_ and *I*_*B*_ are the Pathogen and Beneficial immigration rates; *r*_*P*_ and *r*_*B*_ are the respective maximum per capita growth rates of Pathogens and Beneficials; α is the proportion of substrate taken up by Beneficials and diverted from growth to antibiotic production; *f*(*A*) (which must be a monotonously decreasing function) measures the effect of antibiotics on the Pathogen's net growth rate; *K* is the half-saturation constant for the Monod equation used to limit the growth of Beneficials as a function of nutrient concentration; δ is the decay or mortality rate. We assume a minimum dosage effect in *f*(*A*) (Levin & Udekwu 2010) to scale the effect of antibiotics on *r*_*P*_:

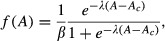


where *A*_*c*_ is the concentration at which antibiotics halve the growth rate, and 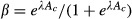 is the normalisation constant. The Pathogen's ‘achieved growth-rate’ terms are the same in the 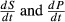 and equations so as to achieve conservation of mass: the material that flows out from the substrate pool flows into bacteria, with a conversion constant that is set to one without loss of generality. [The same holds for Beneficials but is split between allocation to growth (1−α) and to antibiotics (α)].

It is also convenient to assume that δ is the same for all four equations and that the total amount of substrate has reached the stationary value. Thus, antibiotics and Beneficials are produced in a constant ratio, allowing *A* to be substituted by *B*. (See also eqn 8 in Ref. Mao-Jones *et al*. 2010 and Supplement). We redimensionalise, 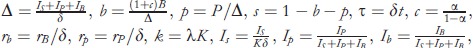 and *b*_*c*_ = *A*_*c*_/α*kI*_*s*_, and achieve a two-dimensional system:

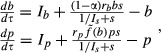
(2)

where 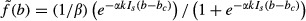. For simplicity, we assume that *I*_*p*_ = 0 and *I*_*b*_ = 0. (Our conclusions do not change if *I*_*p*_ *I*_*b*_ > 0).

Under conservation of mass, *s* = 1−*b*−*p,* and the non-trivial nullclines (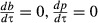) are as follows:

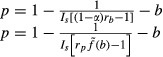
(3)

In Appendix S1, we analyse the basic properties of eqn by studying these nullclines as a function of host resource level *I*_*s*_, the proportion of host resources diverted by Beneficials to antibiotic production α, and bacterial growth rates *r*_*B*_ and *r*_*P*_. We find the following results, recapitulating Mao-Jones *et al*. (2010)

(1) If Beneficials have a higher growth rate, they competitively exclude Pathogens, even when there is no antibiotic production and regardless of the amount of host resources. This might occur if the host provides a substrate that is mainly available to Beneficials (‘private resources’) or simply by chance difference in growth rates. (2) If we assume, more realistically, that Beneficials have a lower growth rate than some Pathogens, Beneficials can never invade a population dominated by those faster-growing Pathogens. (3) However, as Beneficials divert increasing amounts of host resources to antibiotic production, which is a form of interference competition, the community begins to exhibit priority effects (‘bistability’ or ‘alternative stable states’): neither Pathogen nor Beneficial can invade a population already dominated by the other. Instead, there are two stable equilibria: complete dominance by Beneficials or by Pathogens. Which equilibrium is reached depends on which bacterial type has the higher initial abundance. Dominance by Beneficials therefore requires both a sufficiently high per capita production of antibiotics and a sufficiently high initial Beneficial population density, which together create enough total antibiotic to fend off Pathogens.

### Screening-in Beneficials

We now add competition-based screening by the host. Although antibiotic production may confer competitive superiority for the attine-ant niche (Appendix S1), antibiotic production is costly. One possibility therefore is that the host evolves to provide high levels of resources (i.e. higher than in the soil), which can be used to fuel antibiotic production by Beneficials. In fact, it turns out that even when host-supplied resources are assumed to be equally available to both Pathogens and Beneficials (a ‘common-pool resource’), the competitive landscape can still favour Beneficials as long as interference competition is sufficiently strong (i.e. the antibiotics are sufficiently effective).

**Figure 1:**
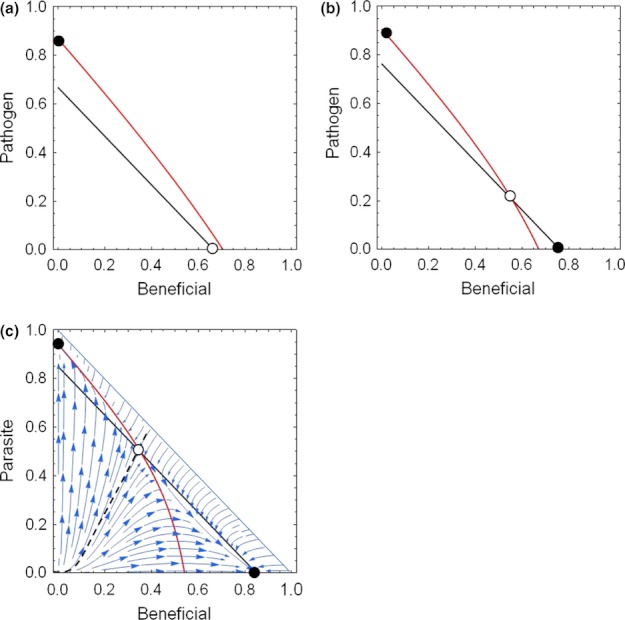
The emergence of bistability as host resource levels *I*_*s*_ increase (eqn 3). The higher the host resource input rate *I*_*s*_, the steeper the Pathogen's nullcline (grey, red online) (A: *I*_*s*_**= 5; B: *I*_*s*_**= 7; C: *I*_*s*_**= 11; black is the Beneficial's nullcline). The system becomes bistable above a critical *I*_*s*_ value. Solid dots indicate stable fixed points, and the empty dot indicates the unstable fixed point (*r*_*p*_**= 2.5, *r*_*b*_**= 2, α = 0.2, *k *= 1, *A*_*c*_**= 0.1). The antibiotic effect of beneficials on pathogens is scaled by 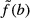, which is a function of 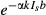. Increasing *I*_*s*_ increases growth rates of all bacteria but also allows greater antibiotic production, leading to suppression of Pathogens.

**Figure 2:**
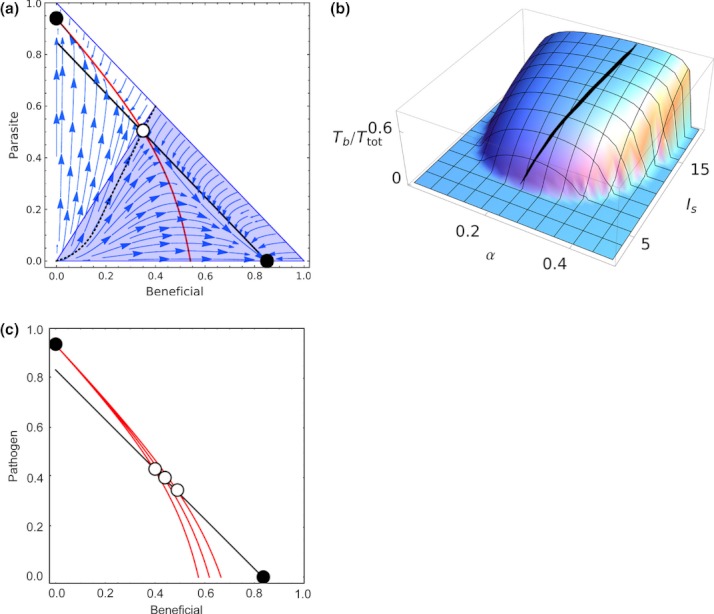
The effect of host resource *I*_*s*_, proportion of resources diverted to antibiotics α, and antibiotic effectiveness *A*_*c*_ on the Beneficial's domain of attraction. (a) The domain (shaded triangle) is approximated as *T*_*b*_**= *p*_*u*_/[2 (*p*_*u*_ + *b*_*u*_)] if 0 < *p*_*u*_, *b*_*u*_ < 1, else *T*_*b*_**= 0 and *P* wins (parameters as Fig. 1). The dashed black curve within the shaded triangle marks the true upper boundary of the Beneficial's domain. (b) The relative domain of attraction (*T*_*b*_/*T*_*tot*_) as a function of α and *I*_*s*_. Increasing α cannot grow the domain above zero when *I*_*s*_ is low, because antibiotic production is insufficient. The black line indicates the optimal α for different *I*_*s*_. (c) The relative domain of attraction is weakly sensitive to antibiotic effectiveness *A*_*c*_ (= 0.1, 0.4, 0.7, l-to-r), suggesting that Pathogen resistance evolution has little effect.

To see this effect of increasing host-provided resources *I*_*s*_ on the dynamics of the microbiome model, again conservatively let Beneficial growth rate be less than Pathogen growth rate, *r*_*b*_ < *r*_*p*_. If there are no Beneficials in the microbiome, *b *= 0, the nullcline of the Pathogen is above the nullcline of the Beneficial, and the Beneficial cannot invade a population of pathogens. However, if Beneficials are present, *b* > 0, increasing host resource provision, *I*_*s*_, steepens the nullcline for the pathogen, eventually causing the system to become bistable (Fig. 1). The Beneficial's domain of attraction increases strongly with host resource provision, *I*_*s*_, but only weakly with the proportion of resources diverted to antibiotic production α and with antibiotic effectiveness *A*_*c*_ (Fig. 2).

### Higher Beneficial immigration rate

**Figure 3:**
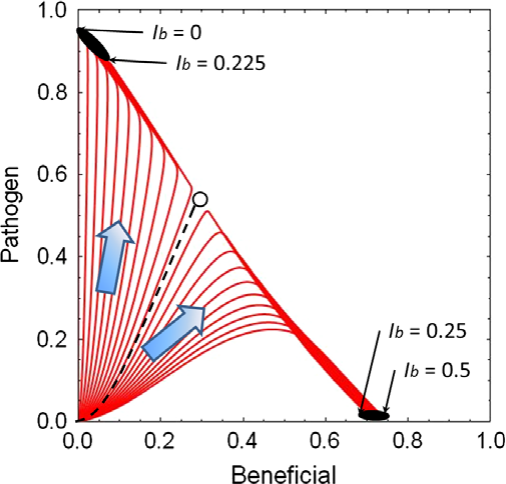
A higher Beneficial immigration rate *I*_*b*_ promotes fixation of Beneficials in a new microbiome in the absence of vertical transmission. Grey (red online) lines denote different community trajectories as *I*_*b*_ changes from 0 to 0.5. The dashed line is the separatrix dividing the two qualitatively different motions denoted by the blue arrows. The open dot is the unstable fixed point, and black dots are the fixed points of the system, which depend on the immigration rate. *I*_*s*_**= 5, *r*_*p*_ = 2.5, *r*_*b*_**= 2, α = 0.2, *k*= 1, *I*_*p*_**= 0.1, *A*_*c*_**= 0.1.

In some systems, it is reasonable to assume that vertical transmission of Beneficials does not occur, such as with plant rhizospheres. However, it is still possible to achieve a higher initial frequency of Beneficials if they have a higher immigration rate from the environment. To model this effect, we assume that the initial density of microbes is zero in the host (no vertical transmission) and assume that there are non-zero immigration rates *I*_*p*_ and *I*_*b*_ in eqn 1. As the behaviour of the system is determined by the initial transient state, we study the original model described in eqn 1. As Fig. 3 shows, if the system is bistable because of high substrate production and effective antibiotic defence, it evolves to the Beneficials-dominated state if the immigration rate of Beneficials is high compared with the immigration rate of Pathogens. So, vertical transmission is not required to achieve a Beneficials-dominated state.

### Three steps to a beneficial microbiome

The effect of bistability is that it is possible for a host to screen-in a microbiome dominated by Beneficials, even if host-provided resources can be consumed by all bacterial types and even if Pathogens have higher intrinsic growth rates. This process can be broken down into three steps: (1) the new host's microbiome starts with a higher proportion of Beneficials, which can be achieved via vertical transmission or via a higher immigration rate of Beneficials due to, for instance, spatial aggregation of previously successful hosts or benign environments (e.g. ‘disease-suppressive’ soils), and (2) the host provides a high resource level, which (3) fuels intense interference competition via antibiotic production and results in competitive dominance by Beneficials.

## DISCUSSION

We have used a phenomenological model to combine stylised facts about one type of microbiome with some simple ideas from game theory (screening) and community ecology (interference competition) to argue that it is possible for hosts to evolve living conditions under which antibiotic-producing bacterial species have a competitive advantage in the host niche.

High general resource availability can favour Beneficials in competition over Pathogens, because even though abundant resources fuel higher growth rates for all bacteria, some of those resources can also be diverted by Beneficials to produce antibiotics (Fig. 2B). There is much circumstantial evidence that hosts provide considerable resources to microbiomes dominated by antibiotic-producing bacteria, such as specialised glands, mucus production, carbon compound export and even a higher frequency of Actinobacteria in the guts of obese humans, relative to lean humans (Ritchie 2006; Goettler *et al*. 2007; Soler *et al*. 2008; White *et al*. 2009; Mendes *et al*. 2011; Seipke *et al*. 2012b).

However, for Beneficials to win, there additionally needs to be a sufficient number of Beneficials at the start of competition, so that enough total antibiotics are produced to kill off faster-growing but susceptible competitors. This problem manifests in the model as bistability, which appears generally in models of species competition with interference competition (Yu & Wilson 2001). Vertical transmission or higher immigration rates can provide the necessary large initial abundance of Beneficials in host offspring.

## Public goods evolution

Although we focused on the problem of partner choice in mutualisms, another evolutionary challenge is social conflict between Beneficials (B) (antibiotic-producers and antibiotic-resistant) and ‘Cheaters’ (C) (non-producers but still antibiotic-resistant). If we allow that B produces a public good for all resistant strains, which is a costly act, then C should always outcompete B, and antibiotic production should disappear. How is cooperation amongst B (antibiotic production) maintained if C has a fitness advantage?

This is the classic problem of public goods evolution, and multiple mechanisms can prevent cheaters from winning over cooperators. The two most likely to be important in microbiomes are as follows

1. Bacteria generally grow on surfaces with restricted dispersal. This limited mixing results in the positive assortment of cheaters and cooperators, meaning that any given bacterial type is more likely to be neighbours with the same type. Consequently, the average benefit of C decreases, and the average benefit of B increases. The strength of this effect depends on the frequency of types, but in general, C does not drive B extinct (Nowak *et al*. 1994). Note that this mechanism works if the public good acts locally, which is more likely in microbiomes like plant rhizospheres: bacteria around a root section protect that section and get their resources from that same, healthy section.
2. A more general mechanism is that if the public good is not a *linear* function of investment, but a *saturating* function of it, then B and C typically coexist, even if the system is well mixed (Archetti *et al*. 2011a; Archetti & Scheuring 2012). Most biological public goods are saturating. For instance, some antibiotic is much better than none, and the marginal benefit of antibiotics then declines with more quantity and/or diversity [1−*f*(*A*) in our model].
Thus, the apparent evolutionary advantage of a C over a B happens only when (1) there is no assortment, and (2) the public good is linear. The absence of both conditions is unlikely in biological systems. As an aside, the parameter governing the level of investment in antibiotic production, α, should be seen as the mean level of investment, allowing some individuals to invest more and others to invest less or nothing.

## Attine microbiomes

Returning to the attines, we conclude that competition-based screening can describe the evolutionary ecology of the attine cuticular microbiome. First, high resource provision by attine hosts is a reasonable assumption, given the presence of specialised glands (Currie *et al*. 2006). Note that ‘high’ is calibrated relative to soil resource availability, not laboratory conditions in which even ‘low-resource’ media are resource-rich in absolute terms.

Second, we can explain high diversity in attine cuticular microbiomes by adding a fourth step to the above scenario. After a Beneficial microbiome is established, other actinomycete species in the soil environment, which typically are resistant to a wide range of antibiotics due to horizontal gene transmission (the ‘resistome’ Wright 2010), should be able to invade and coexist, either neutrally via constant immigration and/or stably via niche partitioning over the host. As a result, we should not be surprised to see signals of both vertical and horizontal transmission in actinobacterial microbiomes, as has been argued for this microbiome (Barke *et al*. 2010; Cafaro *et al*. 2011). Thus, rather than being *competing* explanations, our model suggests that vertical transmission of one bacterial lineage, *Pseudonocardia*, could facilitate horizontal transmission of others, by putting the system in the region of phase-plane space where immigrating Pathogens cannot survive but Beneficials can. This might be *Pseudonocardia*'s most important role in the attine microbiome, and its documented ability to establish dense colonies on the ventral surfaces of some species of attine workers is one way that *Pseudonocardia* can achieve uninterrupted vertical transmission, by facilitating inoculation of new queens, even if other bacteria augment worker microbiomes.

Screening-in additional bacterial species solves the problem of explaining long-term antibiotic efficacy in the attine-ant system: a diverse and constantly changing community of actinomycetes [each with the capacity to produce numerous antibacterials and antifungals simultaneously when in competition with other microorganisms (Challis & Hopwood 2003; Seipke *et al*. 2011)] allows a multi-drug strategy. We can also speculate that *Pseudonocardia* engage in horizontal gene transfer of resistance and antibiotic genes with immigrant actinomycetes. [N.B. We do not suggest that any antibiotic-producing species would be able to invade all microbiomes, as no species is resistant to all antibiotics, even within the *Pseudonocardia* (Poulsen *et al*. 2007).]

Interestingly, the cuticular microbiome in the most derived attine genus, *Atta*, is absent, and behavioural defences such as grooming are more evident in this genus (Mueller *et al*. 2008; Fernández-Marín *et al*. 2009; Cafaro *et al*. 2011), which might suggest that behavioural defences have entirely substituted for antibiotics. However, the example of humans suggests that antibiotics, and the continued recruitment of new antibiotics, remain necessary elements of defence against pathogens. After all, humans also have elaborate behavioural defences against pathogens, from washing to taboos to refrigeration, but the evolution of antibiotic resistance nonetheless poses major clinical challenges. In fact, an alternative explanation for the absence of a cuticular microbiome in *Atta* is given by Mueller *et al*. (2008), who report the ‘consistent presence of [non-*Pseudonocardia*] actinomycete bacteria in gardens of *Atta*…, as well as in queen-pellets of *A. texana*…’. Thus, we hypothesise that the cultivar itself might screen-in a Beneficials-dominated microbiome, as the initial inoculum on pellets appears to be dominated by vertically transmitted actinomycetes. Additional mechanisms for promoting fixation of actinomycetes are (1) stochasticity in the initial microbiome composition such that only incipient cultivars with high-enough Beneficials frequency survive to assemble an actinobacteria-dominated microbiome, (2) spatial aggregation of or simply interactions between successful *Atta* colonies, leading to a higher initial immigration *I*_*b*_ of Beneficials (Fig. 3), and/or (3) antimicrobial secretions by the ant or fungal cultivar (Fernández-Marín *et al*. 2009; Poulsen & Currie 2010), which could impart a competitive advantage to microbes that bear the appropriate resistance genes, as antibiotic-producers are more likely to.

We speculate that one reason for *Atta*'s ecological dominance is that the non-trivial cost of supporting a protective microbiome, if such a microbiome indeed exists in *Atta*, has moved down a trophic level to the cultivar, which would not only increase efficiency but also free *Atta* to invest more in metapleural gland secretions (Poulsen *et al*. 2002; Fernández-Marín *et al*. 2009), therefore making two different kinds of protection available.

## CONCLUSION

Screening can be a somewhat non-intuitive concept. It might help to point out that, unlike with costly signalling, a screening host never needs to detect the qualities of the potential symbionts, nor does the host choose amongst symbionts. Instead, the pool of potential symbionts is placed under selection to colonise or not colonise the host environment, over evolutionary and ecological timeframes, or, under competition-based screening, the symbionts themselves exclude undesired species and genotypes in ecological time (Archetti *et al*. 2011b). Also, while screening in the attine microbiome is straightforwardly described as an ‘action and reaction’ scenario, the fundamental reason to have hosts ‘move first’ in a sequence of interactions is to eliminate the possibility of signalling by the symbiont, which shows that partner choice can be achieved even when the symbiont's characteristics are hidden.

We stress that screening is only one cooperation-enforcement mechanism. Screening can solve the problem of hidden characteristics, but other mechanisms are needed to solve hidden-action and public-goods problems, all of which can arise within a single mutualism (Archetti *et al*. 2011a).

We also emphasise that do not explicitly model host-symbiont coevolution and the origin of antibiotic-producing microbiomes. It remains an open challenge to explain how hosts evolved to provide costly resources to a microbiome. One scenario is that a simple actinobacterial microbiome originated via vertical transmission, and, later, specialised microbial parasites and possibly reduced antibiotic efficacy selected for hosts that provided high levels of resources, which would immediately increase antibiotic production and recruit multiple antibiotic-producing bacteria. In the Supplementary Information, we explore the evolution of a host that reacts to increased Pathogen levels by increasing resource provision, which can be interpreted either as the above scenario or simply as the evolution of increased efficiency in the host. Another origins scenario is that antibiotic-producing but non-mutualistic bacteria had a competitive advantage for the ant niche, which is assumed to have originated as a low-resource environment. However, mutant hosts providing higher resource levels would have resulted in greater antibiotic production, which would be available to use for protection of the ant's fungal cultivar. Vertical transmission could evolve secondarily. We also show in Supplementary Information that the host evolves to an optimal positive resource supply *I*_s_* and that Beneficials evolve to an optimal, positive α* once *I*_*s*_ evolves to a level high enough that the P–B system is bistable.

Testing for screening poses challenges (Archetti *et al*. 2011b). With antibiotic-producing microbiomes, however, one experimental strategy is to follow motion in phase space under qualitatively different conditions (Fig. 1C). Antibiotic-producing (and -resistant) species can be pitted in competition experiments against non-antibiotic-producing (and non-resistant) species, under conditions of high- and low-resource availability and with different starting ratios. For instance, established colonies of Pathogens should not be invasible by Beneficials, and established colonies of Beneficials should be invasible by Pathogens under low-nutrient conditions but not under high-nutrient conditions. As a control, antibiotic production can knocked out in Beneficials (e.g. Seipke *et al*. 2011), which should render them invasible. Another control is that established Beneficials should be invasible by Pathogens that have (or have been engineered to have) appropriate resistance genes. Ideally, such experiments would be done on live hosts, as it is now possible to visualise antibiotic production *in vivo* (Schoenian *et al*. 2011). Attine ants are particularly convenient, as their cuticles can be sterilised. Such a research programme would also constitute a partial test of Read *et al*.'s (2011) argument against aggressive antibiotic treatment, on the basis that the practice only results in the dominance of resistant bacteria in human microbiomes. In both models, resistant types are competitively superior when antibiotics are present, the main difference being that antibiotic production is endogenous in our model.

## Supporting Information

As a service to our authors and readers, this journal provides supporting information supplied by the authors. Such materials are peer-reviewed and may be re-organised for online delivery, but are not copy-edited or typeset. Technical support issues arising from supporting information (other than missing files) should be addressed to the authors.
