# Supplementary material for: How to assemble a beneficial microbiome in three easy steps
Source: Ecol Lett. 2012 Aug 22;15(11):1300–7. doi: 10.1111/j.1461-0248.2012.01853.x (PMC3507015; doi:10.1111/j.1461-0248.2012.01853.x)
Supplement: Supplementary file 1 [file ele0015-1300-sd1.pdf]

How to assemble a beneficial microbiome in three easy steps: Supplementary Information

István Scheuring[1], *Douglas W. Yu*[2,3,*]

[1] Research Group in Theoretical Biology and Evolutionary Ecology, Department of Plant Systematics, Ecology and Theoretical Biology, Eötvös University and HAS, Budapest, Pázmány P. sétány 1/C, H-1117 Hungary. Email: istvan.scheuring@gmail.com
[2] Ecology, Conservation, and Environment Center (ECEC), State Key Laboratory of Genetic Resources and Evolution, Kunming Institute of Zoology, Chinese Academy of Sciences, Kunming, Yunnan 650223 China. Email: dougwyu@gmail.com
[3] School of Biological Sciences, University of East Anglia, Norwich Research Park, Norwich, Norfolk NR47TJ UK
* Corresponding author: tel: +86 1860 871 7369, +44 7510 308 272; fax: +44 1603 592 250; postal address: BIO, UEA, Norwich, Norfolk NR47TJ UK

Reactive hosts

Here we study whether a host that detects and reacts to increasing pathogen level by increasing substrate production has a higher fitness than a nonreactive host. That is, assuming that the host can evolve mechanisms to detect overall pathogen level, is a reactive strategy maintained or not?

We assume that individuals start with a high I_S at the beginning of their life, then I_S decreases to a lower level after the beneficial goes to fixation (e.g. P close to zero). Then if pathogen concentration increases (because of immigration for example), a reactive strategy increases I_S. The conjecture is that this reactive strategy has a higher fitness than a nonreactive one, which is defined as producing a high level of I_S regardless of parasite concentration.

How do we implement this scenario in a simple model?

Let us start with the same model as above. The dynamics lead pathogen's density to be close to zero after a while. Within this initial time interval, I_S takes a high fixed value. We assume

that after a time T, I_S becomes $I_S(P/B)$, such that $I_S(P/B)$ increases with p in a saturating manner. That is, the reactive individual uses the following substrate-producing algorithm:

$$I_S(P/B) = \begin{cases} I_S & \text{if } t < T \\ I_0 + (I_S - I_0)\dfrac{P/(P+B)}{\Theta + P/(P+B)} & \text{otherwise} \end{cases} \qquad \text{(S1)}$$

Parameter Θ is the half-saturation constant, which gives the relative concentration of P, where the concentration-dependent part of substrate production is half of the maximum $(I_S-I_0)/2$. By using this type of function, $I_S(P/B)$ increases with P/B in a Michaelis-Menten-like manner and varies between I_0 and I_S according to the concentration of P.

We compare the above strategy with a nonreactive host producing the same level of $I_S(P/B)=I_S$ for its entire lifetime (e.g. $I_S=I_0$). Thus we study system (1) with the additional assumption of reactive substrate production:

$$\begin{aligned} \frac{dS}{dt} &= I_S(P/B) - \frac{r_P f(A)PS}{K+S} - \frac{r_B BS}{K+S} - \delta S \\ \frac{dB}{dt} &= I_B + \frac{(1-\alpha) r_B BS}{K+S} - \delta B \\ \frac{dA}{dt} &= \frac{\alpha r_B BS}{K+S} - \delta A \\ \frac{dP}{dt} &= I_P + \frac{r_P f(A)PS}{K+S} - \delta P \end{aligned} \qquad \text{(S2)}$$

To compare the relative successes of reactive and nonreactive hosts, we measure their fitnesses as the weighted average of $I_S(t)$ and $P(t)$ so that

$$W = W_0 - a\langle I_S(t)\rangle - \langle P(t)\rangle, \qquad \text{(S3)}$$

where W_0 is a constant independent of the strategy we study here, a is the relative cost of substrate production compared to parasite load, and $\langle . \rangle$ is the time average for the lifetime of the individual. (By using this fitness function, it is assumed that fitness decreases because of increased substrate production or increased parasite infection, and a measures the cost of substrate production in units of cost of parasite infection.)

Numerical studies. - We analyzed system (S2) numerically. We used the same parameter range used before for the nullcline analysis, but our conclusions remain qualitatively valid for

a broader range of parameters. We have two general conclusions according to the simulations:

a) A reactive host is fitter than a nonreactive one, unless substrate production is very cheap (Fig S1, S2).

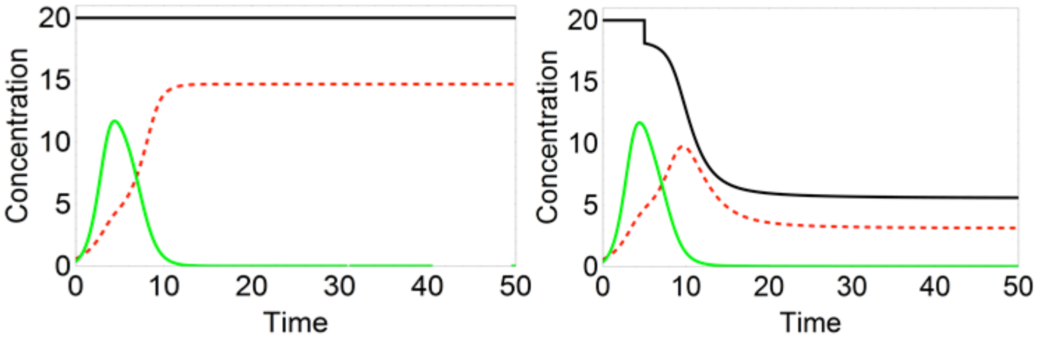

Fig. S1. The time evolution of beneficial (dashed red) and pathogen (green) strains and substrate production rate (black). In the nonreactive model (left hand side), production rate is constant (I_s=10), while in the reactive model, substrate production follows function (S1) with parameters T=5, I_S=15, I_0=5, Θ =0.1. (other parameters r_B=2, r_p=2.5, K=1, α=0.2, δ=1, λ=1. I_P=I_B=0, A_c=0.1, P(0)=0.4, B(0)=0.6.)

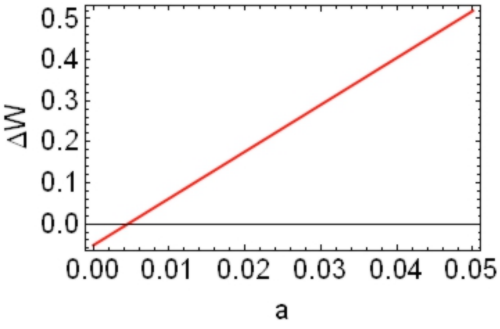

Fig. S2. The fitness difference of reactive and nonreactive hosts, as a function of the relative cost of substrate in eqn. (S3). Positive ΔW indicates a higher fitness for reactive hosts. Parameters are the same as before. Fitness is calculated as time averages from 0 to 50. It can be seen that a reactive host is more fit if substrate cost is above a critical level. This consequence is qualitatively independent on the chosen parameter values.

b) A reactive host defends against pathogen invasion almost as effectively as a nonreactive host

Here we study the effect of parasite invasion. We assume that after the beneficial goes to fixation, the parasite (after a time interval t_P) continuously invades the host with rate I_P (Fig S3, S4).

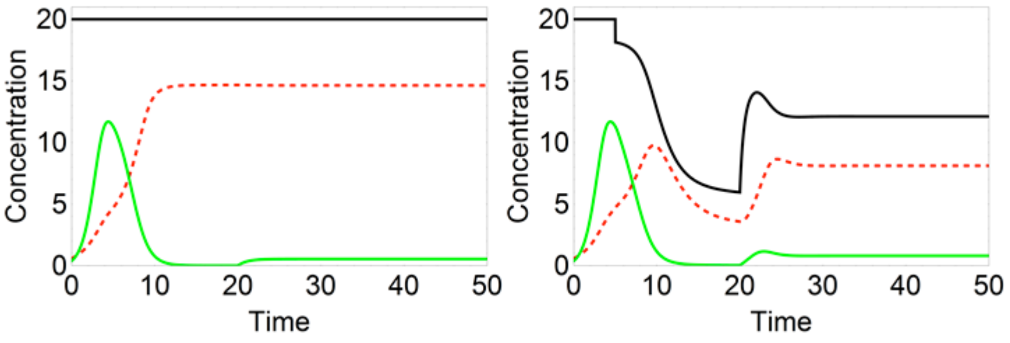

Fig. S3. The effect of parasite invasion in nonreactive and reactive host. Invasion starts at t_P=20 with rate I_P=0.5. Other parameters are the same as before. It is clear that parasite level is higher in the reactive model, but still suppressed with a lower level substrate production.

Again we can compute the fitness of the two host models and compare them as a function of relative cost of substrate production (Fig S4).

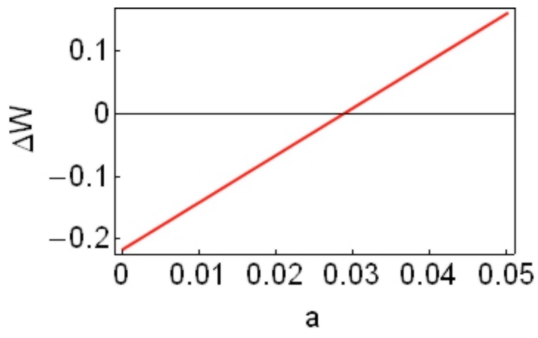

Fig. S4. Fitness difference as a function of the relative cost of substrate production. We use the same dynamical parameters as above. It is clear that constant invasion of parasite causes a significant load to the reactive host, since the critical value of *a* is higher compared to the previous model. In other words, compared to the non-immigration scenario, the substrate has to be more expensive in order to make the reactive strategy fitter. Nonetheless, the trend is the same. It is worth evolving a strategy that reacts to parasite load by increasing substrate production, if substrate is not too cheap compared to the parasite load.

One risk to a reactive host is that Pathogen invasion might occasionally be very intense, leading to successful Pathogen spread within the host. Naturally, such invasions are more likely to be successful in reactive hosts than in nonreactive hosts. This difference simply

causes W_0 to be smaller for reactive hosts than for nonreactive ones, and thus, ΔW will be positive for a larger relative cost a.

Host evolves an optimal I_S

We consider a simple non-reacting host (that is, it produces a constant level of I_S), and we are interested in whether selection among hosts can result in an optimal level of I_S. We define average host fitness based on the assumptions in the **Reactive hosts** section above:

$$\langle W(I_s)\rangle = W_0 - a\langle I_s\rangle - \rho(I_s)\langle P\rangle, \qquad \text{(S4)}$$

where $\rho(I_s)$ is the probability that P wins the competition for a given I_s and $\langle W(I_s)\rangle$ is the average fitness of hosts producing substrate at a rate I_s. Since I_s is constant, $\langle I_s\rangle = I_s$. This is a simple linear model in which the death rate of the host increases with the average concentration of parasites, which is a general assumption. Naturally, whether P or B wins depends on initial conditions. $\rho(I_s)$ measures the fraction of initial conditions when P wins. It generally depends on concentrations of P and B and their invasion rates, etc., but we do not have independent estimates of these quantities. Thus, we consider many hosts with evenly distributed initial conditions of P and B. According to this assumption and our estimated basin of attraction (Fig. 2), we can estimate how $\rho(I_s)$ decreases as I_s increases. Since $\rho(I_s) \approx T_P / T_{tot} = 1 - T_B / T_{tot}$ (if $0<T_P<1$, else $\rho(I_s) = 0$ or 1 respectively), we can approximate $\rho(I_s) = b_u / (b_u + p_u)$. This approximation is very close to the real basin of attraction (Figure 2A). Substituting (4) into this relation gives us

$$\rho(I_s) = \frac{\dfrac{1}{\alpha k}\left[\log\left(\dfrac{1}{\beta}\dfrac{r_p}{(1-\alpha)r_b} - 1\right) + A_c\right]}{I_s - \dfrac{1}{[(1-\alpha)r_b - 1]}}. \qquad \text{(S5)}$$

We are interested in the optimal I_s level, so the question is whether $\dfrac{dW}{dI_s}$ has local maxima.

$$\frac{d\langle W\rangle}{dI_s} = -a - \rho'(I_s)\langle P\rangle = 0$$
$$\rho'(I_s) = -\frac{a}{\langle P\rangle} \qquad \text{(S6)}$$

Substituting (S5) into (S6), $\langle W\rangle$ has a local maximum at

$$I_s^* = \sqrt{\frac{\langle P\rangle}{a}\frac{1}{\alpha k}\left[\log\left(\frac{1}{\beta}\frac{r_p}{(1-\alpha)r_b} - 1\right) + A_c\right] + \frac{1}{[(1-\alpha)r_b - 1]}}$$

since $\left.\frac{d^2\langle W\rangle}{dI_s^2}\right|_{I_s^*} < 0$.

The other solution of (S6) is a local minimum. Note that this analysis conservatively assumes that *P* and *B* concentrations are exogenously determined. A more realistic but more complicated model would allow the concentrations of *P* and *B* to change in the next generation after I_S changes. That is, if I_S increases, then *P* decreases and *B* increases in the next generation, which makes the above dynamic more efficient. Thus, this negative feedback loop between I_S and P intuitively does not change the general conclusion that there exists an optimal I_S; I_S will merely be optimal at a lower level than in our conservative model.

Beneficials evolve an optimal α

Considering the problem as a coevolutionary process, we study not only the evolution of I_s in the host but also the evolution of allocation of resources to antibiotics by the bacteria. Antibiotic resistance is a character of Beneficial bacteria, and thus their average fitness can be estimated as a quantity proportional to the approximated basin of attraction for the B-dominated state, assuming that initial conditions are evenly distributed in [0, 1]. Thus

$$\langle W_B\rangle \propto T_B / T_{tot} = p_u / (b_u + p_u)$$

(More precisely, $\langle W_B\rangle \propto T_B / T_{tot} = p_u / (b_u + p_u)$ if $0 < T_B / T_{tot} \le 1$. Otherwise, Beneficials always lose the competition and $W_B = 0$.)

$$\langle W_B(\alpha)\rangle = 1 - \frac{\frac{1}{\alpha k}\left[\log\left(\frac{1}{\beta}\frac{r_p}{(1-\alpha)r_b} - 1\right) + A_c\right]}{I_s - \frac{1}{\left[(1-\alpha)r_b - 1\right]}} \qquad \text{(S7)}$$

We are interested in whether there are α where $\langle W_B(\alpha)\rangle$ is maximal. The derivative of $\langle W_B(\alpha)\rangle$ is too complex, and there is no way to compute α when W_B is maximal in a closed form, but Fig. 2B (main text) shows that $\langle W_B(\alpha)\rangle$ is flat as a function of α, while for every I_s, there is a maximum point to which α evolves. Thus, if I_s evolves in the host to a level such that the P-B system is bistable, then the Beneficial bacteria evolve to an optimal, positive α^*. Note again that we have assumed conservatively that *P* and *B* concentrations are determined entirely exogenously.
