# Supplementary figures and images for: How to assemble a beneficial microbiome in three easy steps
Source: Ecol Lett. 2012 Aug 22;15(11):1300–7. doi: 10.1111/j.1461-0248.2012.01853.x (PMC3507015; doi:10.1111/j.1461-0248.2012.01853.x)

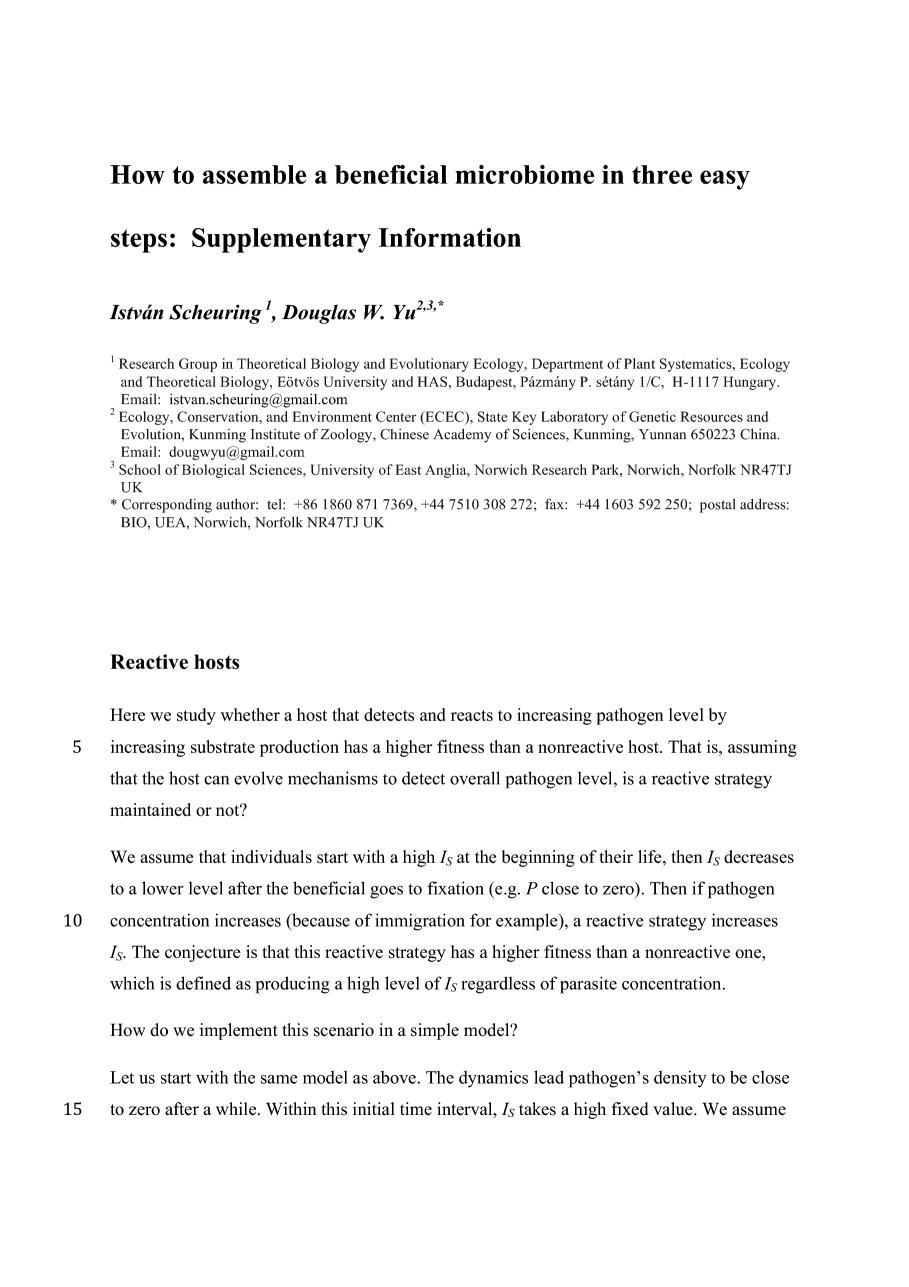

Supplement: Supplementary file 2 [file ele0015-1300-sd2.png]

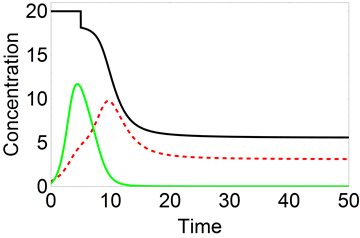

Supplement: Supplementary file 3 [file ele0015-1300-sd3.tif]

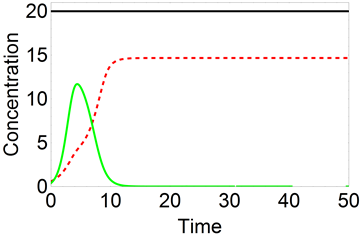

Supplement: Supplementary file 4 [file ele0015-1300-sd4.tif]

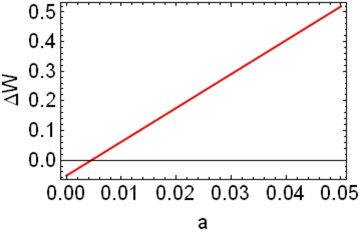

Supplement: Supplementary file 5 [file ele0015-1300-sd5.tif]

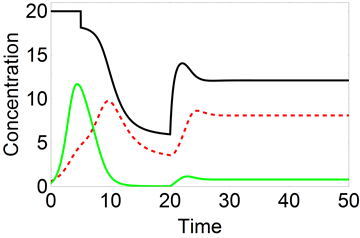

Supplement: Supplementary file 6 [file ele0015-1300-sd6.tif]
